# Simple trachelectomy during pregnancy for cervical cancer

**DOI:** 10.3332/ecancer.2016.673

**Published:** 2016-09-06

**Authors:** Estefania Moreno-Luna, Patricia Alonso, Javier De Santiago, Ignacio Zapardiel

**Affiliations:** Gynaecological Oncology Unit, La Paz University Hospital, Madrid 28046, Spain

**Keywords:** cancer of the cervix, pregnancy, early stage, management

## Abstract

Invasive cervical cancer is rare during a pregnancy, even though it is one of the most frequently diagnosed neoplasias during that time. It is noted that around 30% of women diagnosed with cervical cancer are of reproductive age. This means that up to 3% of cases of cervical cancer are found in pregnant women or those who are in the post-birth period. A cervicovaginal Pap smear is performed as part of the regular checkup for a pregnant woman during the first visit so that cervical cancer can easily be diagnosed early in these women, detecting it early in up to 70–80% of cases. We present here the case of a patient with initial diagnosis of cervical cancer made around 20th week of pregnancy. It was then treated by a simple trachelectomy and cerclage during week 24. The pregnant woman gave birth to a healthy baby at the end of her pregnancy. Definitive treatment was completed three months after giving birth with a total hysterectomy and laparoscopic bilateral salpingectomy while preserving both ovaries. After 17 months of monitoring the patient showed no signs of reoccurrence. In conclusion, during the early stage of cervical cancer conservative management may be a reasonable option to preserve the current pregnancy.

## Introduction

Cancer of the cervix has a global incidence of 500,000 cases per year. Geographical differences are very important, with the incidence rates being especially high in developing countries. Cervical cancer may be considered to have shown a decline in incidence since the 60s, and is currently stable [1]. Invasive cervical cancer is not very frequent during pregnancy, however, it is the most frequently diagnosed cancer along with breast and ovarian tumours during gestation [2]. The incidence of this tumour in women between the ages of 20–49 years is 1.5–14.9/100,000 [3–4], this means that up to 3% of patients are diagnosed during pregnancy [2–5].

In the majority of pregnancy control protocols, a cervical Pap smear is done during the first three months allowing early diagnosis of cervical cancer in women who have not previously been included in a cancer screening programme, therefore up to 70–80% of patients are diagnosed during the early stages [6–8]. It should be noted that in 70–80% of the cases, the tumour histology corresponds to cervical squamous cell carcinoma [9]. Pregnancy does not appear to accelerate the natural history of the tumour or increase the incidence of metastatic disease, although thanks to early diagnosis, the survival rate of patients diagnosed during pregnancy seems somewhat higher than in women who are not pregnant. On the contrary or when there is a late diagnosis, there does seem to be a worse prognosis, partly because of therapeutic limits in these specific cases [10–11].

We present a case of cervical cancer diagnosed during pregnancy in which it was decided to perform a simple trachelectomy and cerclage to preserve the pregnancy.

## Clinical case

We present here the case of a 37-year-old primigravida who went to high-risk obstetrics consultations at our centre because of having a high-grade squamous intraepithelial lesion (SIL) in an opportune cytology screening during the first three months of her pregnancy. There was no medical history of interest.

**Figure 1.**
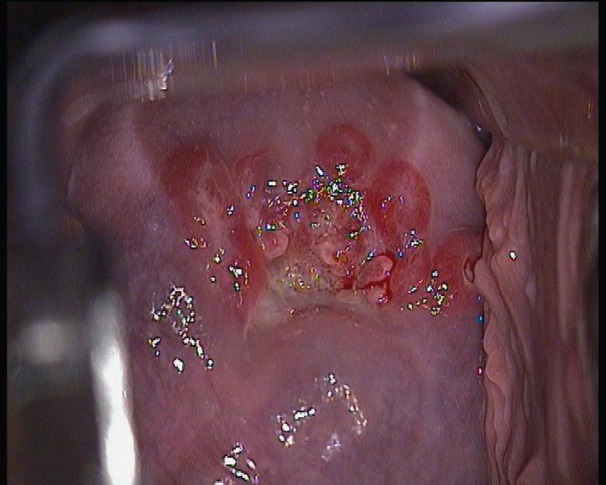
Colposcopic image of the cervical lesion.

A colposcopy was carried out, which showed a 5 mm area with major changes and a biopsy was taken where an invasive epidermoid carcinoma was diagnosed with presence of HPV 16 in week 20 of her pregnancy (Figure 1).

A further study was done by an MRI and a chest x-ray where no evidence was found of distant metastatic disease, nor an affected parametrium. It was found to be compatible with FIGO stage IA1. The Perinatal Medicine Committee and the Oncological Gynaecology Committee jointly decided to perform a simple trachelectomy and cervical cerclage in week 24 of gestation.

The pathological results of the trachelectomy showed micro-infiltrating cervical carcinoma (with 5.5 mm maximum extension on the surface and 1.1 infiltration), with extensive cervical high-grade squamous intraepithelial lesions (HG-SIL), which reach the endocervical glands and is in wide contact with the endocervical margin. There is no evidence of invasion of the lymphovascular space. With this result, it was decided to let the gestation evolve with the appropriate three monthly controls until full term of the pregnancy and allowing a natural vaginal childbirth.

In week 37 of pregnancy the patient began to experience regular uterine contractions, and it was decided to remove the cervical cerclage. After removing the cerclage, the patient went into labour but a caesarean section was finally carried out because of the risk of loss of foetal well-being. A healthy 3030 g girl was born with an umbilical artery of pH 7.19 and Apgar test of 9/9

After giving birth, a new MRI was performed on the patient which was normal. Following three months after birth, the treatment was completed with a total hysterectomy and bilateral laparoscopic salpingectomy while preserving both ovaries with no evidence of remains of disease in the pathological anatomy. The woman was disease-free after 17 months of monitoring.

## Discussion

Although each specific case should be discussed and agreed upon with the patient in an individual way, generally treatment which preserves fertility is an option to consider in pregnant women patients with an initial cervical cancer diagnosis during pregnancy.

An important factor to consider is the gestational period when diagnosing. In general in pregnancies of less than 16–20 weeks consideration should be given to terminating the pregnancy and initiating early cervical cancer treatment, although if the patient wishes to go ahead with the pregnancy, it should be allowed. In those patients where diagnosis occurs later than this gestational period, it appears to be safe to delay treatment until the foetus reaches maturity, and then after giving birth to carry out the definitive treatment without this having a detrimental effect on the patient’s prognosis [12–13].

Patients with stage FIGO IA1–IB1 squamous neck cancer with less than 2 cm tumours and with no lymphovascular invasion may be conservative treatment candidates during gestation with wide conisation or simple trachelectomy, completing the treatment 6–8 weeks after having given birth [6–14].

Some authors consider conisation as a way of ruling out microinvasive disease or for the diagnosis of invasive carcinoma, following which a treatment being necessary after childbirth [5]. In our clinical case the indication of a trachelectomy was intended to establish the final stage and allow the pregnancy to continue with a low cancer risk.

During pregnancy a simple trachelectomy is not exempt of maternal-–foetal complications. It is performed during the second three months of pregnancy preferably and only in patients with strong colposcopic or cytologic evidence of incipient invasive cervical cancer. When the conisation is practised during the first three months, abortion occurs in 33% of cases. Diagnosis in our case was in week 20, and the surgical treatment was given after waiting for a month.

In cases where a conservative treatment is decided during pregnancy, the delivery could be by the vaginal route; caesarean section is reserved for cases in which the tumour stage is greater than IA2, in which case a radical hysterectomy and bilateral pelvic lymphadenectomy could be done during the same surgical treatment. The birth route does not seem to affect the course or spread of the tumour disease, although there is a tendency to avoid the vaginal route in all large tumour cases (greater than 2–4 cms) or in cases in which moderate cervical bleeding is foreseen [15].

It should also be taken into consideration that the treatment options involve an important ethical, emotional, and social development dilemma for the binomial foetus–patient and the medical team. That is why decisions have to be taken by a multidisciplinary team of obstetricians, neonatologists, gynaecologists oncologists, medical oncologists, and pathologists not only in such cases but also in the rest of fertility treatment preservations cases similar to our case. One should always take into account what the patient wants and keeping her opinion central to taking decisions [11–16].

## Conclusion

In conclusion, the therapeutic management will basically depend on the stage of pregnancy when diagnosing the disease, the FIGO stage, the size of the lesion, whether the patient wants to continue with the pregnancy, and also what future reproductive ability is desired by the patient. Treatment with simple trachelectomy and cerclage in week 24 of gestation seems a reasonably safe option to achieve a full term pregnancy in stage IA1 cervical cancer cases during pregnancy.

## Conflict of interests

The authors declare that they have no conflict of interest and have not been financed by any entity.

All authors have revised and approved the final version of the manuscript. All have contributed equally and meet the criteria for authorship.
